# Impact of a 4-Week Intensified Endurance Training Intervention on Markers of Relative Energy Deficiency in Sport (RED-S) and Performance Among Well-Trained Male Cyclists

**DOI:** 10.3389/fendo.2020.512365

**Published:** 2020-09-25

**Authors:** Thomas Birkedal Stenqvist, Monica Klungland Torstveit, Jens Faber, Anna Katarina Melin

**Affiliations:** ^1^Department of Sport Science and Physical Education, Faculty of Health and Sport Science, University of Agder, Kristiansand, Norway; ^2^Department of Medicine, Endocrinology, Herlev University Hospital, Copenhagen, Denmark; ^3^Department of Sport Science, Faculty of Social Sciences, Linnaeus University, Växjö/Kalmar, Sweden

**Keywords:** endurance athletes, energy availability, hormonal response, male cyclists, resting metabolic rate, testosterone, training intervention

## Abstract

Cyclists often apply block periodization to high training volumes in meso- and macrocycles to optimize training adaptation and to prepare for competition. Body mass influences performance in many sports, including endurance disciplines, and conditions related to the syndrome Relative Energy Deficiency in Sports (RED-S) such as metabolic adaptations and premature osteoporosis have also been reported in male cyclists. This study aimed to determine how a 4-week mesocycle of intensified endurance training designed to increase performance, would affect markers of RED-S in well-trained male cyclists. Twenty-two participants (age: 33.5 ± 6.6 years, height: 181.4 ± 5.2 cm, weight: 76.5 ± 7.4 kg, peak oxygen uptake (VO_2peak_): 63.5 ± 6.6 mL·kg^−1^·min^−1^) were recruited and instructed to maintain their background training load and to follow a supervised training protocol consisting of three high-intensity interval training sessions per week with a work duration of 32 min per session. Protocols included pre- and postintervention assessment of resting metabolic rate (RMR) using a ventilated hood, body composition and bone health by dual-energy X-ray absorptiometry (DXA), blood samples, energy intake, and aerobic performance. The interval training increased participants' aerobic performance—peak power output [4.8%, *p* < 0.001], VO_2peak_ [2.4%, *p* = 0.005], and functional threshold power [6.5%, *p* < 0.001] as well as total testosterone levels [8.1%, *p* = 0.011]—while no changes were observed in free testosterone [4.1%, *p* = 0.326]. Bodyweight, body composition, and energy intake were unchanged from pre- to post-test. Triiodothyronine (T_3_) [4.8%, *p* = 0.008], absolute RMR [3.0%, *p* = 0.010], relative RMR [2.6%, *p* = 0.013], and RMR_ratio_ [3.3%, *p* = 0.011] decreased, and cortisol levels increased [12.9%, *p* = 0.021], while no change were observed in the total testosterone:cortisol ratio [1.6%, *p* = 0.789] or the free testosterone:cortisol (fT:cor) ratio [3.2%, *p* = 0.556]. A subgroup analysis of the five participants with the largest increase in fT:cor ratio, revealed a greater improvement in functional threshold power (9.5 vs. 2.5%, *p* = 0.037), and higher relative RMR (0.6 vs. −4.2% *p* = 0.039, respectively). In conclusion, 4 weeks of intensified endurance interval training increased the athletes' aerobic performance and testosterone levels. However, negative changes in markers related to RED-S, such as a reduction in RMR and T_3_, and an increase in cortisol were observed. These results indicate the complexity involved, and that male athletes are at risk of developing clinical indications of RED-S even during a short 4-week endurance training mesocycle.

## Introduction

Preparing for a competitive cycling season often involves high volumes of training, quantified over several periods, ranging over micro-, meso-, and macrocycles, designed to induce specific physiological adaptations (1). If a planned overload is followed by a well-matched recovery strategy, functional overreaching with the intended physiological adaptation occurs (1). However, large training volumes that are combined with insufficient recovery strategies can trigger the development of non-functional overreaching and overtraining syndrome, with symptoms of fatigue and decreased performance (2, 3). Monitoring changes in an athletes' hormone concentrations, including testosterone and cortisol, have previously been used to assess athletes' anabolic-catabolic balance (4). However, monitoring athletes' testosterone-to-cortisol ratio is considered more sensitive to training stress than is merely measuring testosterone and cortisol separately (5).

Several parameters such as body mass and nutritional intake affect cycling performance, and low energy availability is frequently reported among cyclists (6, 7). Energy availability is the amount of energy relative to fat-free mass (FFM) left to support basic body functions after subtraction of the energy used during exercise; energy availability = (energy intake [kcal]—exercise energy expenditure [kcal])/(FFM [kg])/day (8–12). Low energy availability combined with large training volumes, can cause negative consequences such as impaired protein synthesis, impaired hormonal and training response, and increased risk of fatigue; these may lead to performance impairment (5, 9, 10). Research in females has also shown a variety of health parameters being negatively affected by both short- and long-term low energy availability (8–13). Clinical trials exposing eumenorrheic females to low energy availability (<30 kcal·kg^−1^ FFM·day^−1^) for only 5 days found reductions in levels of insulin-like growth factor-1 (IGF-1), leptin, insulin, triiodothyronine (T_3_), and luteinizing hormone (12, 14). Furthermore, long-term low energy availability has been found to increase the risk of premature osteoporosis and to give elevated cardiovascular risk factors (10, 13).

Research reports that male athletes are also vulnerable to the negative health and performance consequences of low energy availability as outlined in the Relative Energy Deficiency in Sports (RED-S) model (10). Not all health and performance aspects of RED-S are, however, fully elucidated, and recent research in male athletes has shown inconsistent findings (15–17). One possible reason for this inconsistency is believed to be the methodological difficulties of assessing energy availability (8, 18). Measurements of resting metabolic rate (RMR), implicating the energy expended on basic bodily functions have therefore been proposed to, and to some extent used as, a potential objective indicator of energy availability (9, 19). However, only two studies to date, have investigated the impact of different training regimens on RMR as a surrogate marker, where one study investigated trained cyclists eliciting overreaching (20), and the other investigated elite rowers during an intensified training period (19). These studies, however, did not include an assessment of hormonal responses when monitoring athlete's responses to changes in energy intake, training regimen or the combination of both.

Therefore, the aim of this study was to determine how a mesocycle of 4 weeks of intensified endurance training designed to increase aerobic performance, would affect RMR, body composition, energy intake, total and free testosterone, cortisol, testosterone:cortisol ratio, T_3_, insulin and IGF-1 levels in well-trained male cyclists.

## Methods

### Study Design

This prospective intervention study was part of a larger training study (clinicaltrials.gov; NCT04075929) with no control group. The training intervention consisted of three supervised high-intensity interval sessions per week, for 4 weeks. Athletes were instructed to maintain their current background training load while enrolled in the study. Each interval training session contained 20 min of self-regulated warm-up, followed by an interval work period with a total accumulated work duration of 32 min of high-intensity training, followed by a 20-min self-regulated cool-down. The total accumulated amount of exercise during the 4-week training intervention was 384 min of high-intensity training and 480 min of self-regulated warm-up and cool-down. Bone mineral density (BMD) was assessed before the intervention, while RMR, body composition, hormone levels, performance variables (peak oxygen consumption and time trial), and energy intake were assessed before and after the intervention period.

### Participants

**Figure 1:**
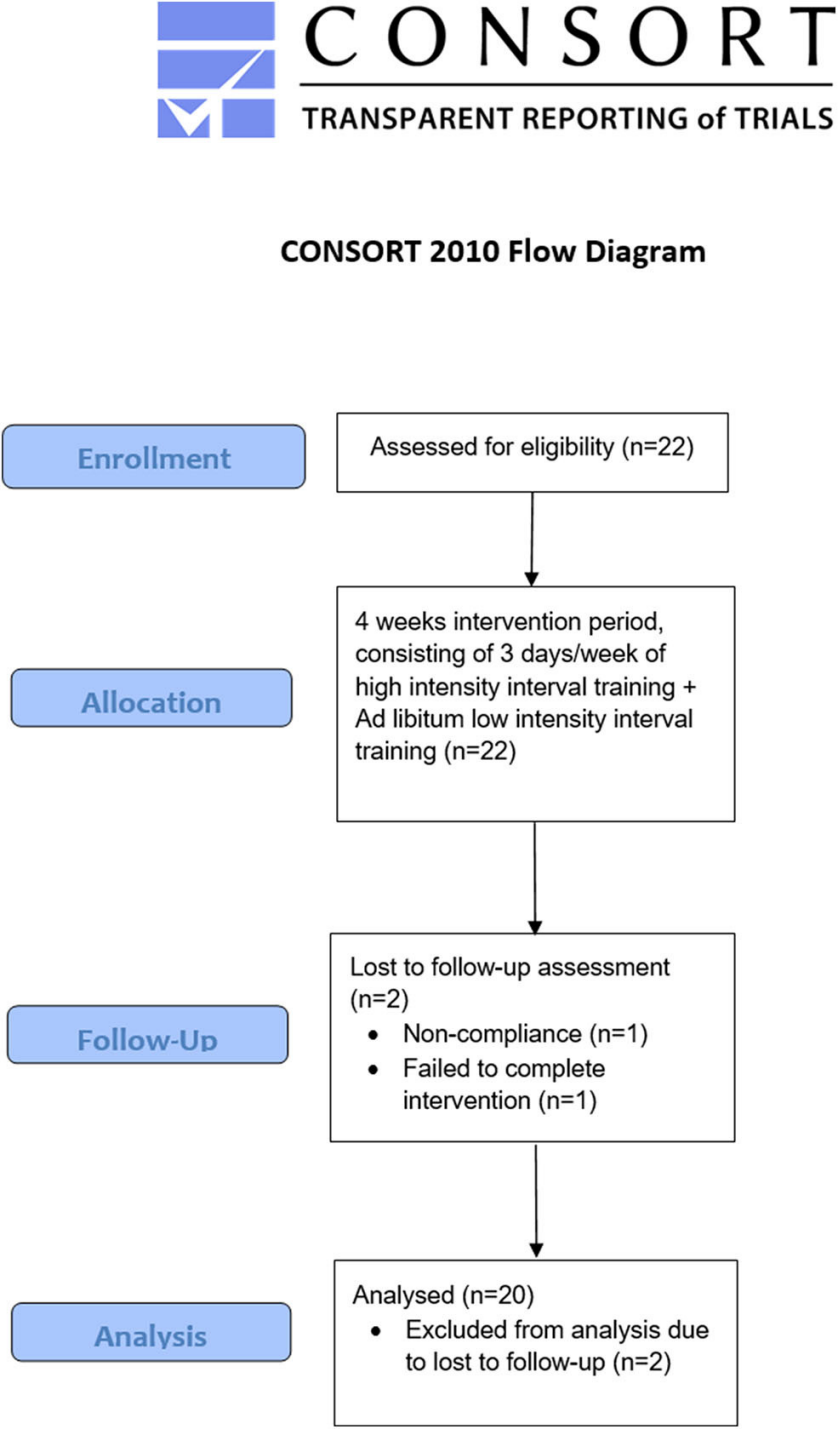
Participation flow. The study was conducted in Kristiansand, Norway.

To be included in the study, participants had to be at least 18 years old but younger than 50 years, with a peak oxygen uptake (VO_2peak_) ≥ 55 mL·kg body mass^−1^·minutes^−1^ and a training frequency of at least three sessions per week during the last year. Furthermore, absence of disease and injury was required. All participants' were classified at performance level 3–4 (21). Recruitment was accomplished through social media and local online newspapers. Before inclusion, all participants received information about the study and test procedures, and signed an informed consent agreement. The study protocol was approved by the University Faculty Ethics Committee and the Norwegian Center for Research Data (no. 46706). All testing complied with the Declaration of Helsinki. Initially, 22 well-trained male cyclists aged between 22 and 45 years who competed at a regional or national level were recruited (Figure 1). Throughout the intervention, two participants were excluded from the analysis: one failed to complete the intervention, and one was excluded because of non-compliance; hence 20 participants were included in the final analysis.

### Procedures and Measures

**Figure 2:**
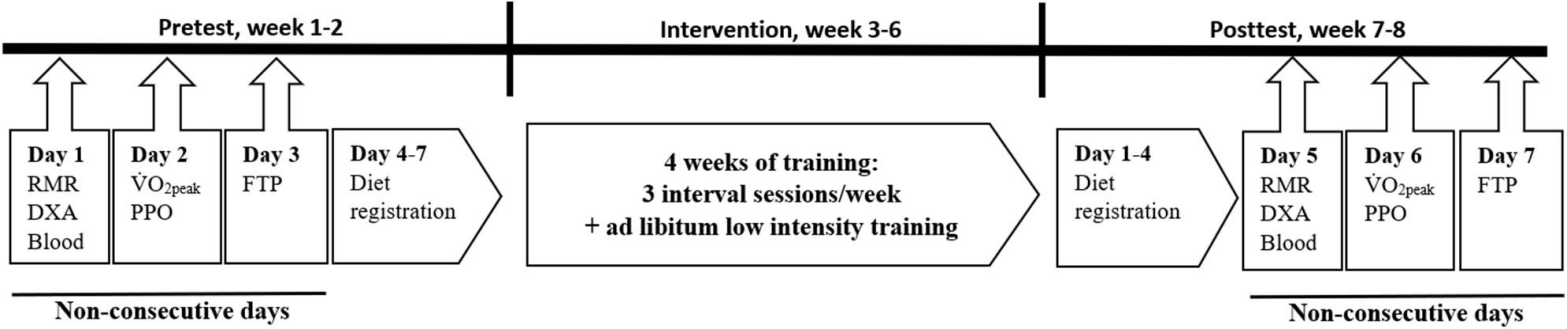
Schematic overview of study design. Pre- and posttest measures included rested and fasted resting metabolic rate (RMR), dual-energy X-ray absorptiometry (DXA), and blood sampling. An incremental exercise test for determination of peak oxygen uptake (VO_2peak_) and peak power output (PPO) as well as a 40-min functional threshold power-test (FTP) were performed in an unfasted state. Diet registration were performed during four consecutive days.

Training volume from the last 4 weeks before pretesting, as well as during the intervention, was collected via written training diaries. During a 2-week period before and after the intervention, participants completed physiological testing, and logged their dietary intake. Participants arrived at the university laboratory during three non-consecutive days for physiological testing. On the first day, participants arrived using motorized transport in a fasted and rested state. RMR, body composition, BMD, and blood sampling were assessed at 06:00–09:00 a.m. to control for diurnal variation. On the second day, participants completed a maximum aerobic exercise testing protocol at 12:00–05:00 p.m. in an unfasted state. On the third test day, the participants performed a 40-min time trial to assess their functional threshold power. During the last week before, and just after the intervention, participants weighed, and registered their dietary intake for four consecutive days (Figure 2).

### Performance Variables

VO_2peak_ and peak power output measurements were performed on a stationary bike (Excalibur Sport, Lode B.V., Groningen, the Netherlands) starting with 1 min of cycling at a power output of 175 W and increased by 25 W per minute until voluntary exhaustion or failure to maintain a cadence of at least 70 rounds per minute. Oxygen consumption (VO_2_) and carbon dioxide production (VCO_2_) was measured using Oxycon Pro™ with mixing chamber and 30 s sampling time (Oxycon, Jaeger GmbH, Hoechberg, Germany) and was calibrated according to standard laboratory procedures. Mean power during the last minute of the test decided the 'cyclist's peak power output, and the mean from the two highest VO_2_ measurements determined VO_2peak_. Heart rate was measured continuously, and blood lactate was measured 1 min after test completion. Objective criteria, such as plateau of the oxygen uptake, heart rate ≥ 95% of known heart rate peak, respiratory exchange ratio ≥ 1.10, and blood lactate ≥ 8.0 mMol·L^−1^ were used to ensure a valid test (22). To assess functional threshold power, participants performed a seated 40-min all-out test using their own bike on CompuTrainer Lab bike rolls (Race Mate, Seattle, WA, USA). The test started with a 20–30-min warm-up at a self-regulated load, followed by a 40-min ride with the highest possible mean wattage. All participants were blinded for power output and heart rate, with only time remaining and rounds per minutes displayed.

### Resting Metabolic Rate

Indirect calorimetry using a canopy hood system was used to assess RMR (Oxycon Pro, Jaeger, Germany), and systems were calibrated before each test according to standard laboratory procedures. Participants rested for 15 min before measurement. VO_2_ and VCO_2_ were assessed over a 30-min period. The last 20 min of measurements were used to assess RMR as described elsewhere (23). Measured RMR was calculated using the Weir equation (24) (3.94 × VO_2_ [ml]) + (1.1 × VCO_2_ [ml]) × 1.44. Relative RMR was calculated as measured RMR in kcal·kg^−1^ FFM·day^−1^. Predicted RMR was calculated using the Cunningham equation (25) (500 + (22 × FFM [kg]), and RMR_ratio_ was calculated as measured RMR [kcal]/predicted RMR [kcal]. Resting heart rate (V800, Polar Elektro Oy, Kempele, Finland) was defined as the lowest heart rate measured during RMR measurement.

### Energy Intake and Macronutrients

Participants weighed and registered their dietary intake for four consecutive days using a digital kitchen scale (OBH Nordica 9843 Kitchen Scale Color, Taastrup, Denmark). In-depth oral and written instructions were given before registration, and participants were asked to maintain their habitual dietary patterns and routines during the registration period. All dietary data were logged using software from Dietist Net (Dietist Net, Kost och Näringsdata, Bromma, Sweden) with access to the Norwegian food table and an open Norwegian nutritional information database.

### Body Composition and Bone Mineral Density

Height was measured without shoes to the nearest 0.1 cm using a wall-mounted centimeter scale (Seca Optima, Seca, Birmingham, UK). Body weight was measured in underwear to the nearest 0.01 kg with an electronic scale (Seca 1, model 861, Birmingham, UK). Body mass index (BMI) was calculated as weight in kg divided by height squared in meters (kg/m^2^). Body composition and BMD were assessed using dual-energy X-ray absorptiometry (DXA) (GE-Lunar Prodigy, Madison, WI, USA, EnCore software version 15). The same technician performed all tests with the same scanner on all participants. BMD was assessed in the lumbar spine (L1–L4), femoral neck, and total hip. Low BMD in athletes was defined as a Z-score of < −1.0 in one of the measured sites, as recommended by Nattiv et al. (11). Body composition was assessed according to a best-practice protocol (26), including assessment of hydration status (USG) before DXA measurement using a digital refractometer (Atago UG-α cat. no. 3464, Atago U.S.A. Inc., Bellevue, WA).

### Blood Sampling

Blood samples were drawn from the cephalic vein of participants 5 min after completion of DXA. Two 5 mL Vacuette Z Serum Sep clot activators (BD, Plymouth, UK) were filled and centrifuged at 3,100 × g for 10 min (Statspin Express 4, Beckman Coulter, Inc. 250 S. Kraemer Blvd. Brea, CA, USA) within the limit of at least 30 min but <60 min. Five 2 mL Cryotube vials (VWR International, Radnor, Penn, USA) were filled with serum and frozen to −80°C. Serum was analyzed at Sørlandets Hospital, Kristiansand and analyzed for testosterone (analytic CV: 6.7 %), sex hormone-binding globulin (SHBG 4.0%), T_3_ (6.9%), cortisol (8.2%), insulin (21.1%), and IGF-1 (8.0%). Free testosterone was calculated by dividing total testosterone with SHBG.

### Statistics

Data were analyzed using Statistical Package for the Social Sciences (SPSS) for Windows (v. 25; IBM Corp., Armonk, NY, USA). The dataset was controlled for missing data and signs of non-normality using histograms as reference, and the assumption of normality of variance was found to be satisfied. Difference and relative changes between pre- (PRE) and posttest (POST) were assessed using paired-samples *t*-test (POST-PRE), generating mean, standard deviation of difference, and 95% confidence interval including percent change. Changes between groups were found using independent sample *t*-test. Effect size (ES) was calculated to interpret the meaningfulness of results using Cohen (27) criteria (0.2 = small effect, 0.5 = medium effect, 0.8 = large effect). Statistical significance level was defined as *p* < 0.05. A priori power analysis was calculated based on an expected standard deviation of 2.0 (19), and we had 80% power to detect a true mean group difference at 1.9 kcal·kg^−1^ FFM·day^−1^ in relative RMR with a minimum of 11 participants (alpha: 0.05; two-tailed).

## Results

Descriptive characteristics of the participants are presented in Table 1. Comparing total accumulated training load (including background training) from before pretesting with total accumulated training during the intervention, no significant increase was found (*p* = 0.497).

**Table 1:** Descriptive characteristics of athletes included in the final analysis.

**Variables**	**All (*n* = 20)**
Age (years)	33.3 ± 6.7
Height (cm)	180.8 ± 4.9
Weight (kg)	75.8 ± 7.3
BMI (kg/m^2^)	23.2 ± 1.9
Body fat (kg)^†^	11.1 ± 4.5
Body fat (%)^†^	14.9 ± 5.2
FFM (kg)^†^	65.5 ± 5.2
Resting HR (beats/minute)	48.0 ± 8.0
VO_2peak_ (mL.kg^−1^.minute^−1^)	63.5 ± 6.6
VO_2peak_ (L.minute^−1^)	4.8 ± 0.4
Exercise (h/year)	395 ± 171
Active within cycling (years)	12.9 ± 9.7

*Data are presented as mean ± SD*.

† *measured by DXA. BMI, body mass index; DXA, dual-energy X-ray absorptiometry; FFM, fat-free mass; HR, heart rate; VO_2peak_, peak oxygen uptake*.

### Performance

Athletes improved their VO_2peak_ (2.4%, *p* < 0.01), peak power output (4.8%, *p* < 0.001) and functional threshold power (6.5%, *p* < 0.001) from pre- to post-test (Table 2).

**Table 2:** Aerobic performance variables at pre- and posttest. Results from paired-sample *t*-tests (post-pre).

**Outcome measure**	**Pre**	**Post**	**Mean ± SD of difference**	**95% CI**	***P*-value**	**Δ Post-Pre (%)**	**ES**
PPO (Watt)	397	416	18.5 ± 12.4	12.7–24.3	<0.001	4.8	1.49
VO_2peak_ (mL.kg^−1^.minute^−1^)	63.5	65.0	1.5 ± 2.1	0.5–2.5	0.005	2.4	0.72
VO_2peak_ (L.minute^−1^)	4.8	4.9	0.1 ± 0.2	0.01–0.2	0.026	2.1	0.54
FTP (Watt)	261	278	17.0 ± 11.8	11.5–22.5	<0.001	6.5	1.44
FTP (Watt/kg)	3.5	3.7	0.2 ± 0.2	0.2–0.3	<0.001	6.9	1.48

*Data are presented as mean ± SD of difference, 95% CI, percent change from pre to posttest and effect size (Cohen's D). PPO, aerobic peak power output; VO_2peak_, peak oxygen uptake; FTP, functional threshold power*.

### Resting Metabolic Rate, Energy Intake, Body Composition, and Bone Health

A 3.0% reduction was found in both absolute RMR, relative RMR, and RMR_ratio_ from pre- to post-test (*p* < 0.05) (Table 3). No changes were observed in energy intake (kcal/day) or intake of macronutrients (g per kg, E%). Body weight, fat mass, and FFM was assessed during stable urine specific gravity (0.001 ± 0.006 kg.m^3^, *p* = 0.420), and did not differ from pre- to post-test (Table 3). L1-L4 average Z-score was 0.1 ± 1.1, while average femoral neck and total hip Z-scores were 0.2 ± 1.0 and 0.3 ± 0.9, respectively. Three athletes (15%) had low BMD in either L1-L4, femoral neck, or total hip, respectively: participant 1 (Z-scores: −3.6, −2.2, and −2.0, 26 years old, 17.4% fat); participant 2 (+0.2, −1.1 and −1.1, 36 years old, 15.9% fat); and participant 3 (−1.8, +1.2, and +0.9, 43 years old, 24.7% fat).

**Table 3:** RMR, energy intake, macronutrients, and body composition at pre- and posttest. Results from paired-sample *t*-tests (post-pre).

**Outcome measure**	**Pretest**	**Posttest**	**Mean ± SD of difference**	**95% CI**	***P*-value**	**Δ Post-Pre (%)**	**ES**
Absolute RMR (kcal·day^−1^)	1,768	1,716	−52 ± 81	−90.3 to −14.1	0.010	−3.0	0.64
Relative RMR (kcal·kg^−1^ FFM·day^−1^)	26.9	26.2	−0.8 ± 1.2	−1.3 to −0.2	0.013	−2.6	0.67
RMR_ratio_	0.91	0.88	−0.03 ± 0.04	−0.1 to 0.0	0.011	−3.3	0.75
Energy intake (kcal)	3,015	3,021	5.6 ± 560.6	−256.7 to 268.0	0.965	0.2	0.01
Carbohydrate intake (g)	332	338	5.2 ± 74.2	−30.6 to 40.9	0.766	1.8	0.07
Relative carbohydrate intake (g/kg)	4.4	4.5	0.1 ± 1.1	−0.4 to 0.6	0.607	2.2	0.09
Protein intake (g)	124	128	4.7 ± 25.0	−7.4 to 16.7	0.428	3.2	0.19
Relative protein intake (g/kg)	1.6	1.7	0.07 ± 0.4	−0.1 to 0.2	0.388	6.3	0.17
Fat intake (g)	123	125	1.7 ± 29.8	−12.6 to 16.1	0.803	1.6	0.06
Relative fat intake (g/kg)	1.6	1.7	0.03 ± 0.5	−0.2 to 0.2	0.741	1.8	0.06
Body weight (kg)^†^	75.8	75.7	−0.16 ± 0.7	−0.5 to 0.2	0.342	−0.1	0.23
FFM (kg)^†^	65.5	65.5	−0.05 ± 0.8	−0.4 to 0.3	0.764	0.0	0.06
Fat mass (kg)^†^	11.1	11.0	−0.09 ± 0.7	−0.4 to 0.2	0.563	−0.9	0.13

*Data are presented as mean ± SD of difference, 95% CI, percent change from pre- to post-test and effect size (Cohen's D)*.

† *measured by DXA. DXA, dual-energy X-ray absorptiometry; FFM, fat-free mass; RMR, resting metabolic rate*.

### Blood Markers

Total testosterone increased 8.1% (*p* = 0.011) from pre- to post-test while no significant changes in free testosterone (4.1%, *p* = 0.326) were found. Cortisol levels increased 12.9% (*p* = 0.021) while total testosterone:cortisol ratio (1.6%, *p* = 0.789), and free testosterone:cortisol ratio (−3.2%, *p* = 0.556) remained unchanged from pre- to post-test. Mean T_3_ levels decreased 4.8% (*p* = 0.008) while no significant changes were observed in insulin and IGF-1 levels from pre- to post-test (Table 4).

**Table 4:** Blood markers at pre- and posttest. Results from paired-sample *t*-tests (post-pre).

**Outcome measure**	**Pretest**	**Posttest**	**Mean ± SD of difference**	**95% CI**	***P*-value**	**Δ Post-Pre (%)**	**ES**
Total testosterone (nmol/L) [7.2–24.0]	17.4	18.8	1.35 ± 2.13	0.35–2.35	0.011	8.1	0.63
Free testosterone (nmol/L) [0.168–0.607]	0.459	0.478	0.020 ± 0.087	−0.021 to 0.060	0.326	4.1	0.23
Cortisol (nmol/L) [138.0–690.0]	381.1	430.3	49.25 ± 87.31	8.39–90.11	0.021	12.9	0.56
Free testosterone:cortisol ratio	0.00125	0.00121	0.0001 ± 0.0003	−0.0001 to 0.0001	0.556	−3.2	0.06
Total testosterone:cortisol ratio	0.047	0.046	0.001 ± 0.012	−0.007 to 0.005	0.789	1.6	0.06
SHBG (nmol/L) [8.0–60.0]	39.7	40.2	0.45 ± 4.23	−1.53 to 2.43	0.640	2.8	0.11
T_3_ (nmol/L) [1.2–2.8]	2.1	2.0	−0.12 ± 0.18	−0.02 to −0.04	0.008	−4.8	0.67
Insulin (pmol/L) [≤160.0]	34.7	31.0	−3.70 ± 10.20	−8.48 to 1.08	0.121	−10.6	0.36
IGF-1(nmol/L) [17.0–63.0]	18.1	18.0	−0.16 ± 2.06	−1.12 to 0.81	0.740	−0.6	0.08

*Data are presented as mean ± SD of difference, 95% CI, percent change from pre to post-test and effect size (Cohen's D). [xx-xx] indicates reference values. IGF-1, insulin-like growth factor-1; SHBG, sex hormone-binding globulin; T_3_, triiodothyronine*.

A subanalysis that included the five participants with the largest increase and the five participants with the largest decrease in their free testosterone:cortisol (fT:cor) ratio from pre- to post-test revealed quantitatively quite similar changes in both total and free testosterone. In the group with an increased fT:cor ratio, there was a pronounced increase in testosterone (19 and 25%, total and free testosterone, respectively) and a 7% decrease in cortisol. This contrasted the group with decreased fT:cor ratio, where there was a decrease in total and free testosterone of 4 and 8%, respectively, combined with a 32% increase in cortisol. Furthermore, a greater improvement in functional threshold power was observed in the high fT:cor ratio group vs. the low fT:cor ratio group (9.5 vs. 2.5%), and similarly a higher relative RMR (0.6 vs. −4.2%, respectively) (Table 5). No differences were found when comparing training volume before or during the intervention in the high vs. low fT:cor ratio subgroups (*p* = 0.609).

**Table 5:** Relative changes in hormonal and performance variables from pre- to post-test in participants with highest increase vs. highest decrease in free testosterone:cortisol ratio. Results from independent sample *t*-test.

**Relative change (%)**	**Highest increase (*n* = 5)**	**Highest decrease (*n* = 5)**	***P*-value**
Total testosterone	18.7 ± 20.6	−4.1 ± 8.9	0.053
Free testosterone	24.7 ± 26.0	−7.8 ± 9.0	0.030
Cortisol	−6.9 ± 17.7	32.3 ± 15.5	0.006
SHBG	−4.1 ± 6.1	4.2 ± 7.4	0.088
T_3_	−10.0 ± 3.5	−11.0 ± 7.4	0.803
Insulin	−8.1 ± 17.6	−1.3 ± 30.0	0.675
IGF-1	−7.2 ± 3.0	−3.9 ± 6.8	0.352
PPO	6.3 ± 4.1	4.2 ± 3.2	0.388
VO_2peak_	3.8 ± 3.8	2.4 ± 3.8	0.557
FTP	9.5 ± 5.4	2.5 ± 3.1	0.037
Training volume (h/week)	−5.1 ± 31.5	2.7 ± 18.9	0.650
Energy intake	6.6 ± 22.6	10.2 ± 38.2	0.860
Relative RMR	0.6 ± 2.8	−4.2 ± 3.3	0.039
Body weight	−0.4 ± 0.7	−0.5 ± 1.2	0.901
FFM	−0.7 ± 1.1	0.3 ± 0.6	0.126

*Data are presented as mean ± SD. FFM, fat free mass; FTP, functional threshold power; IGF-1, insulin like growth factor-1; PPO, peak power output; relative RMR (kcal/kg FFM/day), resting metabolic rate; SHBG, sex hormone-binding globulin; T_3_, triiodothyronine; VO_2peak_, peak oxygen uptake*.

## Discussion

In this study, we have demonstrated that 4 weeks of high-intensity training for 32 min, three times a week, superimposed on the athletes' background training, resulted in increased aerobic peak power output, VO_2peak_, functional threshold power, as well as increased testosterone levels. In contrast, markers associated with low energy availability such as decreased RMR, lowered T_3_, and increased cortisol, were found. Thus, our findings suggest positive performance responses of the exercise program used in the present study, however negative responses related to health, potentially caused by lowered energy availability were observed. This is a worrying sign, that a relative short period of 4 weeks can induce such changes, and athletes need to take this seriously.

### Resting Metabolic Rate, Energy Intake, Body Composition, and Bone Mineral Density

In the present study, cyclists undertook a 4-week intensified endurance training intervention, which without any apparent increase in their energy intake, led to reduced energy availability, and a 3% reduction in RMR. This is similar to the findings of other studies (19, 20); indeed a 5% decrease in RMR was reported by Woods and co-workers (19) when elite male and female rowers undertook 4 weeks of heavy endurance training, without dietary compensation. Meanwhile, the same group reported that male cyclists achieved a state of overreaching and reduced RMR when 6 weeks of intensified training was undertaken without adjustment of energy intake (20). Meanwhile, other studies of increases in training workloads in endurance-trained male cyclists (28) or healthy males undertaking resistance and endurance training (29) reported an increase, or no change in RMR, respectively. However, in these studies, energy intake was either not assessed (28), or measured using a suboptimal protocol of a 3-day recall (29), and it is uncertain whether energy compensation accounted for the divergent results. RMR is mostly affected by body composition, with FFM as the largest determinant accounting for up to 70% of the individual variation in RMR, and is considered one of the largest components of total energy expenditure (30). In the present study, body composition, including FFM, remained unchanged from pre- to post-test and was therefore unlikely to contribute to the reduced RMR, despite several RED-S-related hormonal indications of a more catabolic state. It is unclear whether the increase in testosterone levels, possibly triggered by the endurance interval training, acted as a protective mechanism to prevent increased proteolysis. Hence, the lowered RMR might be a protective mechanism to prevent weight reduction and changes in body composition. Similar findings have been reported in elite male endurance athletes with low energy intake compared with athletes with adequate energy intake, where RMR was calculated to be 8% lower in the low energy intake group, suggesting an energy-conserving mechanism for maintaining body function and stable body weight (31).

Poor bone health develops over a long period with several influential factors, where a lack of loading due to the mode of exercise and poor nutrition are key factors (8, 10, 11). It is well-documented that long-term low energy availability is linked to poor bone health in both male and female athletes (8, 10, 11, 13), and road cycling does not induce significant osteogenic benefits compared with weight-bearing sports (32). Olmedillas et al. (33) reported lower BMD in young cyclists compared with recreationally active age-matched controls, and a recent Norwegian study showed that as many as 53% of elite cyclists had low BMD in the lower extremities, despite reporting regular resistance training (34). In our study 15% of the athletes had low BMD in either the lumbar spine, femoral neck, or total hip. Despite not having information on our athletes previously athletic history, it still raises concerns of athletes being unaware of the potentially negative effects of the lack of bone-loading involved in non-weight-bearing exercise, not performing high-load exercise that dampens the effects of bone loss as well as the intake of insufficient amounts of macro- and micronutrients.

The etiology of low energy availability is complex and may include excessive exercise, “making weight” before a competition, eating disorders, or unintentional mismatch between energy expenditure and energy intake resulting from a lack of appetite, poor nutrition knowledge, or lack of time to plan and prepare meals (11). In the present study, carbohydrate intake was lower and protein intake was higher than recommended (35, 36) and remained unchanged from pre- to post-test. In weight reduction periods, a higher protein intake at the expense of carbohydrates has been shown to improve the amount of fat loss and to preserve lean tissue (37), and this may contribute to the explanation of maintained FFM, despite unchanged energy intake during the intensified endurance training period in the present study. Nonetheless, the importance of periodizing energy intake to make changes in nutritional demands during different phases of training has previously been demonstrated (19); this should be emphasized to help support and enhance endurance training adaptations, especially when athletes undergo strenuous meso- and macrocycle training (38).

### Blood Markers

We observed an increase in total testosterone levels from pre- to post-test, presumably as a positive response from the intensified endurance training protocol. We measured testosterone directly, and calculated free testosterone as well, by dividing total testosterone with SHBG. Based on the old free hormone hypothesis, free testosterone should be the bioavailable form of testosterone. However, this hypothesis has been debated for three decades, without definite conclusion. Thus, a recent comprehensive review by Goldman et al. (39) concluded that no measure of testosterone is ideal, and that both total and free testosterone should be considered. The calculated free testosterone, found by dividing total testosterone with SHBG, is also hampered by assumptions of association constants, and further accuracy and precision are affected negatively by the need for using two analyses. In the literature on the effect of training on testosterone levels, most studies have reported only total testosterone levels. Therefore, we chose to report both measures. The increase in total testosterone observed, could partly be a result of an observed small, however insignificant increase in SHBG. Testosterone, an anabolic steroid, stimulates growth, increases protein synthesis, and controls the development and maintenance of the secondary sex characteristic. Previous studies have demonstrated acute changes in testosterone in resistance training and high volumes of exercise using large muscle mass (40). Severe reductions in testosterone have been reported in male soldiers undergoing prolonged starvation (41), while Koehler et al. (16) found no reduction in testosterone when males were exposed to short periods of very low energy availability (~15 kcal·kg^−1^ FFM·day^−1^) for 4 days.

In the present study, cortisol increased by 12.9% from mean values of 381–430 nmol/L. Cortisol is likely to contribute to increased adiposity during energy abundance, and is an important catabolic hormone secreted to ensure glucose homeostasis during prolonged exercise, glycogen depletion, stress, and starvation (13, 42). A meta-analysis of human studies by Nakamura et al. (43) investigating fasting and severe caloric restriction found increased cortisol levels followed by a long-term normalization, while another study by Kyrolainen et al. (17) found increased cortisol as a response to heavy prolonged physiological stress in soldiers, followed by an immediate reduction when soldiers experienced a stress reduction. However, in a study comparing nine long-distance male runners with low energy availability with eight non-athletes with optimal energy availability, cortisol was not different between the groups (44). The increase in cortisol in our athletes could, therefore, be a combination of a natural response to a sudden increase in high-intensity training as well as an increased need to catabolize alternate energy sources and preserve glycogen, as shown previously (17).

Increased training volumes, combined with insufficient recovery strategies, increases the risk of non-functional overreaching (2, 3). A decrease in the testosterone:cortisol ratio of 30% has been suggested as an indicator of poor recovery (45, 46) and a catabolic status (4, 5), while a value of 0.35 × 10^−3^ has been suggested as a threshold of overtraining (46). In the present study 50% of the participants increased their free testosterone:cortisol ratio during the intervention period, while 50% had a reduced free testosterone:cortisol ratio, including two athletes who had a decrease of > 30%. In the exploratory subanalysis where we looked at the five athletes with the highest increase and five athletes with the greatest decrease from pre- to post-test values of the fT:cor ratio, the changes in total and free testosterone were quantitatively similar, and the ratio was not affected by changes in SHBG to a major degree. Of particular interest, we also found a greater improvement in functional threshold power in those with the highest fT:cor ratio increase compared with those with the largest decrease. Due to a combined increase in free testosterone and decrease in cortisol levels and maintained RMR, this indicates a highly improved anabolic state from pre- to post-test in our study. Although no differences were found in the changes in energy intake between the groups, a > 4% reduction in RMR indicates low energy availability in the participants with the largest decrease the in fT:cor ratio (19). Interestingly, none of the participants in this group showed any signs of preexisting low energy availability on endocrine markers, body composition, BMD, or markers related to RMR; hence, the changes in RMR could potentially be linked to inadequate recovery in this group. Unfortunately, no information regarding heredity of low BMD, history of earlier eating disorder behavior, or what type of training the athletes did before starting being active within cycling were available. Furthermore, when examining the training diaries from before the intervention with the diaries from during the intervention, we found no differences between subgroups. Unfortunately, we do not know the exact distribution of their low- and high-intensity training before the intervention, due to low compliance regarding intensity distribution.

IGF-1 is a pro-insulin-like structure with broad anabolic properties, and low levels are linked to starvation and chronic undernutrition (47). Insulin is a metabolic hormone involved in energy balance, and insulin secretion is correlated with visceral fat in humans, and particularly in males (48). In an eight-week military-exercise study with extreme starvation, Friedl et al. (41) reported a 50% reduction in both IGF-1 and insulin, suggesting improved insulin sensitivity, with a normalization of IGF-1 after a refeeding period halfway through the intervention; however, IGF-1 returned to its declining trajectory when energy again was restricted. Research by Koehler and co-workers (16) showed no reduction in IGF-1, while a decrease in insulin of 36% was observed during short periods of severe low energy availability. These results are similar to the findings in the present study, although we found a non-significant decrease in insulin of 11%. It is possible that the athletes' energy deficiency was not large enough to initiate significant changes, or that the athletes in our study were able to refeed and recover in the week between the last exercise bout and testing.

We did, however, observe a reduction in T_3_, an important hormone for growth, reproduction, and metabolism (13), and a suggested surrogate marker of low energy availability, widely associated with suppressed RMR (49, 50). However, in the study by Koehler et al. (16) where they exposed males to very low energy availability, they found no reduction in T_3_, and therefore questioned whether exercising men are more robust to short bouts of low energy availability compared with sedentary and exercising women. A reduction in T_3_ among soldiers experiencing prolonged starvation has been reported (41), and a recent study reported lower T_3_ levels in males with testosterone levels within the lowest quartile of the reference range compared with males with testosterone levels above this threshold (51).

In the present study, the intensified endurance training protocol could potentially have induced the increase in testosterone levels, while subclinical low energy availability could have induced the lowered RMR and T_3_ and increased cortisol levels. Although indications of low energy availability with an increased catabolic state and a less positive response of the training intervention were found in a subgroup of participants, it is possible that the intensified mesocycle superimposed on their habitual training load, was not strenuous enough to induce widespread hormonal changes. Other reasons could be that the participants' overall energy deficit was not large enough to induce the severe endocrine changes associated with clinical low energy availability in males earlier reported (16, 41). Unfortunately, we were not able to obtain a thoroughly detailed training load from the participants' habitual training pattern, or energy availability, since the details of the participants' training diaries were of inadequate quality to distinguish between high-intensity and low-intensity training.

### Limitations

To minimize limitations in this study, we used strict best-practice protocols developed for RMR and body composition assessments, including urine specific gravity tests to secure reliable results for comparison (26, 52, 53). Furthermore, we had an appropriate number of participants to gain sufficient statistical power, and we used a 4-day consecutive dietary record period mirroring participants typical food patterns, including weighed dietary records to assess energy intake. Although most assessments in the present study were performed in a controlled laboratory-based setting, some limitations must still be acknowledged. First, the sample was classified as having some convenience sample characteristics. Second, we acknowledge that RED-S is a complex field of research, and the results presented in this study should be interpreted with care, given that: (1) various individual responses to intensive exercise occurs, (2) the cyclists were not matched according to training/hormonal status, (3) the study design did not include measurement of energy intake, exercise energy expenditure, and changes in RMR during the intervention period, only pre- and posttest, (4) we experienced low compliance regarding the details of training diaries, making it difficult to assess total accumulated high- and low-intensity training prior to and during the intervention, and (5) we had no control group. We acknowledge that the lack of a control group makes it difficult to conclude with certainty that the changes are due to the intervention. We therefore also chose a more exploratory approach, by investigating the participants with the highest increase/decrease in fT:cor ratio, aiming at generating new hypotheses. Regarding the analysis of testosterone, it should be emphasized that this analysis has inherent and until now unsolved problems, as discussed earlier. Finally, we also acknowledge that the participants in this study were free-living, well-trained athletes, not elite athletes. This makes them prone to stresses outside of our control, including those associated with obligations to family and friends, work and study loads, as well as lifestyle factors that may have influenced their training load.

## Novelty Statement

Periods with increased training loads are common as part of an attempt to increase aerobic performance. Today, only a few studies have examined how various intensified endurance training regimens expose male athletes to the risk of RED-S, and this study contributes to new knowledge on a group of athletes not previously investigated; it also uses blood markers, as called for in recent studies (19, 20). The present study demonstrated that 4 weeks of high-intensity endurance training superimposed on their regular training increased athletes' aerobic performance and testosterone levels. However, adverse changes in markers related to low energy availability, such as a reduction in RMR and T_3_, and an increase in cortisol were observed. It is, however, unclear whether these changes resulted from a lack of increase in energy intake *per se*, if the length of the intervention period was too short to identify more severe clinical changes in markers of low energy availability, or a combination of both. It is worrying, that negative changes in RED-S-related parameters were observed after only 4 weeks of intensified endurance training, and our findings substantiate the importance of further understanding and monitoring RED-S in male athletes undertaking intensified endurance training regimens, as well as increased awareness and education among athletes and coaches.

## Practical Implications

The present study indicates that well-trained male athletes seem to underestimate the importance of matching their energy intake when undertaking a mesocycle of intensified endurance training. This is challenging, and practitioners should be aware that male athletes are also prone to develop indications of RED-S even during a short intensified 4-week endurance mesocycle. Investigating and understanding RED-S, especially in male athletes, is a complex and difficult task. Several markers exist to help researchers and practitioners to interpret energy availability among athletes. The use of blood markers as one of several measures should be included in future research to better understand how males respond to various levels of endurance exercise regimens in combination with assessing their RMR and energy availability. Furthermore, to prevent RED-S-associated conditions in athletes, established and available tools, such as the RED-S clinical assessment tool (RED-S CAT), may be of value for practitioners and health personnel (8, 54).

## Data Availability Statement

The datasets generated for this study are available on request to the corresponding author.

## Ethics Statement

The studies involving human participants were reviewed and approved by University Faculty Ethics Committee and the Norwegian Centre for Research Data (No. 46706). The patients/participants provided their written informed consent to participate in this study.

## Author Contributions

The study was conceptualized and designed by TS, MT, and AM. Data were collected and analyzed by TS. Contribution were made to materials analysis by TS, MT, JF, and AM. Visualization was performed by TS. Writing of the original draft was performed by TS. Reviewing and editing were done by TS, MT, JF, and AM. All authors approved the final version of the paper.

## Conflict of Interest

The authors declare that the research was conducted in the absence of any commercial or financial relationships that could be construed as a potential conflict of interest.
